# Pro-Dermcidin as an Emerging Regulator of Innate Immunity in Sepsis

**DOI:** 10.3390/ijms26157643

**Published:** 2025-08-07

**Authors:** Li Lou, Jianhua Li, Weiqiang Chen, Cassie Shu Zhu, Xiaoling Qiang, Haichao Wang

**Affiliations:** 1 The Feinstein Institutes for Medical Research, Northwell Health, 350 Community Drive, Manhasset, NY 11030, USA; llou@northwell.edu (L.L.); jli@northwell.edu (J.L.); wchen6@northwell.edu (W.C.); czhu3@northwell.edu (C.S.Z.); xqiang@northwell.edu (X.Q.); 2 Department of Emergency Medicine, Donald and Barbara Zucker School of Medicine at Hofstra/Northwell, 500 Hofstra Blvd., Hempstead, NY 11549, USA

**Keywords:** anti-inflammatory, anti-microbial, innate immune cells, pro-dermcidin

## Abstract

Human dermcidin (DCD) is synthesized as a 110-amino acid precursor (pre-dermcidin, pre-DCD) containing a 19-residue leader signal sequence, which is removed to produce a leader-less pro-domain-containing peptide termed as pro-dermcidin, pro-DCD. Pro-DCD can be secreted by human eccrine sweat glands and then cleaved into antimicrobial peptides, such as dermcidin (DCD). Emerging evidence suggests that pro-DCD has broader physiological roles beyond antimicrobial defense, potentially serving as a therapeutic agent for inflammatory diseases like sepsis. In this review, we summarize recent evidence supporting pro-DCD as a regulator of innate immunity in sepsis.

## 1. Introduction

Sepsis, a life-threatening condition characterized by a dysregulated host response to infection, frequently results in organ dysfunction and mortality. Despite advances in critical care, effective treatments targeting the underlying pathophysiology remain limited [1]. Current clinical treatment is largely supportive, consisting primarily of antibiotics and fluid resuscitation [2,3,4]. Therefore, identifying novel protective proteins capable of eradicating invading pathogens and moderating sepsis-induced inflammation remains crucial [5]. Dermcidin (DCD), an antimicrobial peptide secreted by eccrine sweat glands [6,7], and its precursor, pro-dermcidin (pro-DCD), may hold such promise. While DCD has established antimicrobial activity [6,8], emerging evidence suggests that pro-DCD plays a broader immunoregulatory role during sepsis [9]. Here, we review recent evidence supporting pro-DCD as a regulator of innate immunity in sepsis.

## 2. Dermcidin as a Sweat Gland-Derived Antimicrobial Peptide

**Figure 1:**
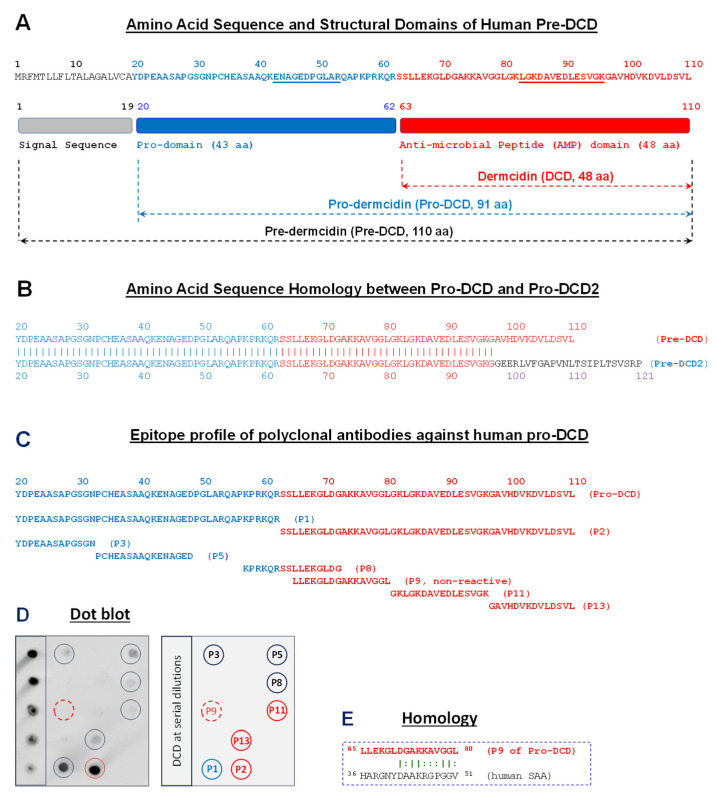
Amino acid sequence of human pro-dermcidin (pro-DCD) and epitope profile of anti-pro-DCD polyclonal antibodies. (**A**) Amino acid sequence and structural domains of human dermcidin precursor, pre-dermcidin (pre-DCD). (**B**) Amino acid sequence homology between human pro-DCD (20–110) and pro-DCD2 (20–121) isomer. (**C**) Sequence of synthetic peptides corresponding to various regions of human pro-DCD. (**D**) Epitope mapping of human pro-DCD-specific polyclonal antibodies by dot blotting analysis of recombinant pro-DCD and thirteen synthetic peptides (P1–P13) corresponding to different regions of pro-DCD. (**E**) Depiction of a peptide sequence (P9) that exhibits some homology to an inducible human acute-phase protein, serum amyloid A (SAA). Reprinted with permission from [9]. Copyright CC-BY, version 4.0.

Humans and other primates have evolved eccrine sweat glands that can produce sweat as an evaporative heat loss mechanism of thermoregulation. In addition, these eccrine sweat glands also secrete various antimicrobial peptides, including pre-dermcidin (pre-DCD) [6,7,10], a 110-amino acid precursor with a 19-residue leader signal sequence (Figure 1A). Following the removal of this signal sequence, the leader-less pro-domain-containing dermcidin (pro-DCD) can be secreted by the eccrine sweat glands and enzymatically processed to produce the mature form dermcidin (DCD, Figure 1A), which exhibits bactericidal activity against various pathogens such as *Escherichia coli*, *Enterococcus faecalis*, and *Staphylococcus aureus* [11,12].

Beyond eccrine sweat glands [6,7,11], emerging evidence suggests that pro-DCD may be upregulated and/or secreted by innate immune cells, such as human monocytes [13], in response to human immunodeficiency virus (HIV) infection [13] or *E. coli* exposure [14]. In rodents, pro-DCD expression has been detected in footpad sweat glands [15], peripheral blood mononuclear cells (PBMCs) [15], and models of myocardial ischemia/reperfusion [16]. Recently, we generated some evidence to support its therapeutic potential in animal models of inflammatory diseases [9,17], supporting its role as an endogenous regulator of innate immunity in sepsis.

## 3. Pro-DCD as an Inducible Protein

To identify endotoxin-inducible proteins, human PBMCs were stimulated with bacterial lipopolysaccharide (LPS) for 16 h, and the culture medium was analyzed by mass spectrometry. This revealed a marked increase in extracellular levels of a 12 kDa protein, which was identified as human pro-DCD by in-gel trypsin digestion and mass spectrometry analysis [18]. Despite the existence of other pro-DCD isomers (Figure 1B) [19,20], the detection of a unique peptide (97-GAVHDVKDVLDSVL-110) confirmed its identity as human pro-DCD (Figure 1B). Immunoblot analysis using a commercial polyclonal antibody (Cat. #ab157719, Abcam, Cambridge, MA, USA) specific to residue 96–110 of human pro-DCD further confirmed dose-dependent pro-DCD secretion by LPS-stimulated PBMCs [18]. Furthermore, real-time RT-PCR analysis showed increased *Dcd* mRNA expression in PBMCs of some donors after LPS stimulation (0.5 µg/mL) [18], suggesting that bacterial endotoxins may induce *Dcd* expression and pro-DCD secretion in human PBMCs. Our findings extended previous reports of pro-DCD upregulation in human PBMCs or bone marrow-derived mesenchymal stromal cells (BM-MSCs) in response to HIV infection [13] or *E. coli* exposure [14]. Furthermore, our findings are consistent with earlier reports of circulating pro-DCD in patients with various diseases, including ischemic stroke [14], facioscapulohumeral muscular dystrophy (FSHD) [21], melanoma [22], and obstructive sleep apnea [23].

## 4. Pro-DCD as a Protective Protein in Sepsis

To investigate the extracellular role of pro-DCD in sepsis, we generated some polyclonal antibodies (pAbs) against human pro-DCD and characterized their epitope profile by dot blotting using 13 synthetized peptides (“P1–P13”) spanning different regions of the protein (Figure 1C). Pre-immune rabbit serum showed no reactivity against recombinant pro-DCD, confirming the absence of pre-existing pro-DCD-reactive autoantibodies in these rabbits [9]. In contrast, anti-pro-DCD serum revealed a strong band at the expected molecular weight of recombinant pro-DCD [17,24]. To obtain antigen-affinity purified IgGs, we first isolated total IgGs from anti-pro-DCD rabbit serum using Protein-A-affinity chromatography, followed by pro-DCD-antigen-affinity chromatography to enrich for antigen-specific IgGs. Dot blot analysis confirmed that these anti-pro-DCD pAbs reacted with recombinant pro-DCD and recognized 7 out of 13 synthetic peptides spanning different regions of pro-DCD (Figure 1D), validating their specificity [9]. Although sepsis also induces hepatic expression and systemic accumulation of another 12 kDa protein, serum amyloid A (SAA) [25,26], our anti-human-pro-DCD pAbs did not react a peptide (e.g., P9) that shares some homology with human SAA (Figure 1E), further supporting the possibility of pro-DCD induction in rodents during severe infections.

To explore the functional role of pro-DCD in sepsis, we examined the impact of antigen affinity-purified anti-pro-DCD IgGs on sepsis-induced systemic inflammation and tissue injury. Administering these pro-DCD-specific IgGs significantly increased sepsis-induced systemic accumulation of granulocyte colony stimulating factor (G-CSF), interleukin 6 (IL-6), and monocyte chemoattractant protein 1 (MCP-1)—three established surrogate markers of experimental [27,28] and clinical sepsis [29]. Furthermore, these anti-pro-DCD antibodies worsened sepsis-induced liver injury and dose-dependently increased sepsis-induced animal lethality [9], suggesting a potentially protective role for pro-DCD in experimental sepsis.

## 5. Therapeutic Efficacy of Pro-DCD and Derivatives in Sepsis

To evaluate pro-DCD’s therapeutic potential, we generated highly purified recombinant pro-DCD (residue 20–110, Figure 1A) with an N-terminal 6×His tag [17,24]. Since pro-DCD can dimerize via a disulfide bond at Cys34 (Figure 1A), we also generated a monomeric pro-DCD mutant with a Cys (C)→Ser (S) substitution at this position. To extend its half-life, we chemically conjugated water-soluble polyethylene glycol (PEG) to the Lys (K) residue of pro-DCD-C34S. This PEGylation was expected to shield pro-DCD-C34S from enzymatic degradation and to reduce its renal clearance. Administering recombinant pro-DCD at 2 h and 24 h post cecal ligation and puncture (CLP) significantly enhanced animal survival from 10% in the saline control to 60% in the pro-DCD-treatment group [9]. When the first dose was delayed to 24 h post-CLP, pro-DCD-C34S showed a similar trend, improving survival from 40% to 70% at the same dose (0.5 mg/kg) [9]. Notably, PEG-pro-DCD-C34S conferred significant protection against lethal sepsis even with delayed administration (24 h post-CLP) at a molar concentration almost 5-fold lower than pro-DCD-C34S [9]. Collectively, these findings indicate that both pro-DCD and pro-DCD-C34S derivatives confer significant protection against lethal sepsis, with PEGylation further enhancing the therapeutic potential of pro-DCD-C34S.

**Figure 2:**
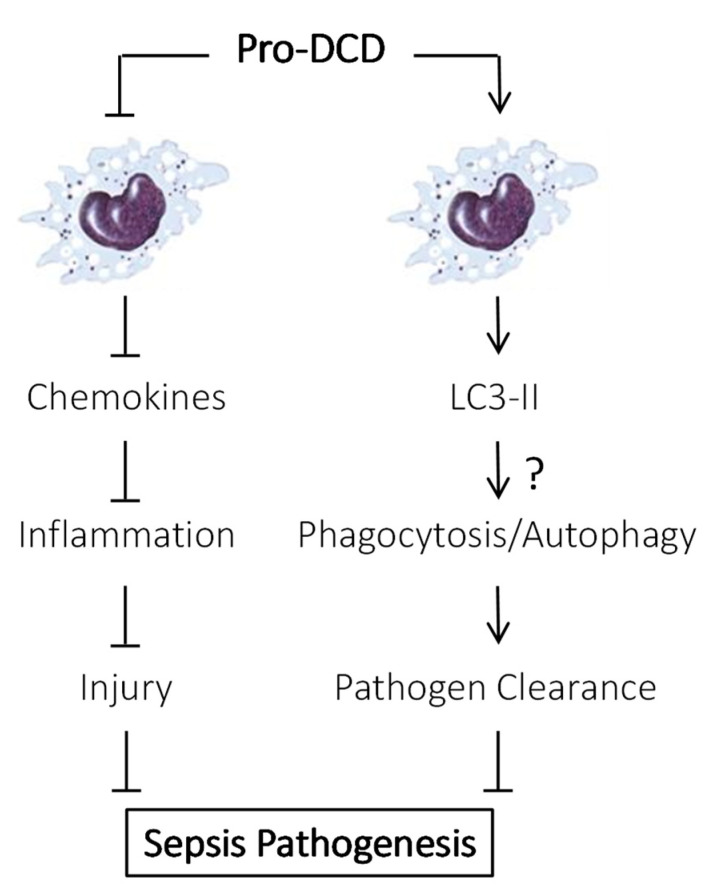
Proposed role of pro-DCD in regulating innate immunity in sepsis. In response to severe bacterial infections, innate immune cells may upregulate and secrete pro-DCD into the bloodstream. Extracellular pro-DCD may confer protection against lethal sepsis partly by attenuating sepsis-induced dysregulated inflammatory injury and partly by facilitating systemic bacterial clearance possible via microtubule-associated protein 1A/1B-light chain 3 (LC3)-mediated phagocytosis and/or autophagy-dependent pathogen destruction. It remains to be determined whether pro-DCD loses its protective effects when LC3 is pharmacologically inhibited or genetically disrupted in future independent studies (“?”).

Therefore, our studies have suggested a protective role for pro-DCD upregulation and secretion in a bacterial infection model (Figure 2). While antigen-affinity-purified pro-DCD pAbs exacerbated sepsis-induced systemic inflammation and tissue injury, recombinant pro-DCD-C34S and its PEGylation derivatives conferred significant protection when administered at 24 h post-CLP. These findings align with previous reports that systemic administration of pro-DCD-C34S mitigated hepatic ischemia–reperfusion injury [17] and highlight the need for future studies evaluating the clinical potential of pro-DCD-C34S and its derivatives in various inflammatory diseases.

## 6. PEG-Pro-DCD-C34S Reduced Sepsis-Induced Inflammation and Bacterial Dissemination

To understand the mechanisms underlying pro-DCD-mediated protection, we evaluated the effects of PEGylated pro-DCD-C34S (PEG-proDCD-C34S) on sepsis-induced inflammatory injury and bacterial dissemination. Intraperitoneal administration of PEG-pro-DCD-C34S markedly reduced systemic levels of G-CSF, IL-6, keratinocytes-derived chemokine (KC), MCP-1, macrophage inflammatory protein-2 (MIP-2), and soluble tumor necrosis factor receptor I (sTNFRI)—surrogate markers of sepsis [29,30,31]. Additionally, PEG-pro-DCD-C34S significantly mitigated sepsis-induced elevation in liver enzymes such as aspartate aminotransferase (AST) and alanine aminotransferase (ALT) [9], suggesting its protective effects extend to reducing both inflammation and tissue injury. This finding is consistent with our previous reports indicating that pro-DCD attenuated KC/GRO-α production in innate immune cells stimulated with exogenous bacterial endotoxins (e.g., LPS) or endogenous mediators of lethal endotoxemia and sepsis (e.g., high mobility group box 1, HMGB1) [1,24,32]. It is also consistent with our recent report that pro-DCD or pro-DCD-C34S conferred protection against hepatic ischemia–reperfusion partly by reducing hepatic MIP-2/GRO-β expression [17].

To further elucidate pro-DCD’s protective mechanism, we examined its impact on bacterial clearance in a murine sepsis model. As expected, CLP led to substantially increased blood bacteria counts due to cecal bacterial spillage into the blood stream [9]. However, PEG-pro-DCD-C34S treatment nearly eradicated detectable bacteremia [9], suggesting that pro-DCD’s protection against lethal sepsis involves enhanced bacterial clearance. Thus, the pro-DCD-C34S-mediated protection can be attributed to attenuated inflammation, reduced tissue injury, and enhanced bacterial clearance (Figure 2).

## 7. Pro-DCD Induced LC3 Activation in Innate Immune Cells

The mechanisms by which pro-DCD facilitates bacterial elimination remain an intriguing subject for future investigation. It has been proposed that pro-DCD’s C-terminal antimicrobial domains (residue 63–110, Figure 1A) can form hexameric structures that interact with bacterial phospholipids, causing membrane depolarization and bacterial death [33,34,35]. These findings corroborate recent reports demonstrating dermcidin’s bactericidal activity [8,12] and its role in counter-regulating innate immunity [36] and microbial dysbiosis in both healthy individuals [37] and patients with diabetes [38]. To understand how pro-DCD facilitates bacterial clearance in septic animals, we incubated pro-DCD or PEG-pro-DCD-C34S with *E. coli* BL21 strain for a few hours before plating the mixture onto LB broth agar at serial dilutions to determine colony-forming units. However, in contrast to the reported antibacterial activities of C-terminal antimicrobial peptide (AMP) domains (Figure 1A) [6,11,39], we found that pro-DCD and its PEGylation derivatives did not directly kill *E. coli*, even at high concentrations (up to 200 µg/mL) [9]. It remains elusive whether the presence of pro-domain in pro-DCD physically hinders the formation of hexameric structures by its antimicrobial peptide (AMP) (Figure 1A), which might be essential for bacterial membrane disruption and subsequent cell death [33,34,35].

To explore alternative mechanisms, we examined pro-DCD’s effect on LC3 (microtubule-associated protein 1A/1B-light chain 3) activation in macrophage cultures. Consistent with a previous report showing that DCD-containing extracellular vesicles promoted LC3-associated phagocytosis of *E. coli* by macrophages [14], we found that pro-DCD significantly increased LC3-II production and LC3 punctum formation in macrophage cultures [7]. This suggests that pro-DCD may promote bacterial elimination through LC3-associated phagocytosis and a special form of autophagy termed “xenophagy” (Figure 2)—two highly conserved pathways used by phagocytes to degrade and clear extracellular and intracellular bacteria, respectively [40,41,42].

During phagocytosis, engulfment of extracellular bacteria triggers LC3-II recruitment to the single-membrane phagosome, facilitating lysosomal fusion, rapid acidification, and bacterial destruction [42]. In contrast, xenophagy sequesters intracellular bacteria within a double-membrane autophagosome, which also fuses with lysosomes to degrade the pathogen [43,44,45]. It is possible that pro-DCD may enhance bacterial clearance by facilitating LC3-associated phagocytosis and the autophagic degradation of engulfed pathogens (Figure 2). This is consistent with prior findings that various autophagy-inducing agents—including antibiotics, endotoxins, bedaquiline, seriniquinone, and antibacterial peptides—can enhance antimicrobial responses [46,47,48,49,50,51]. However, future independent studies are needed to determine whether pro-DCD’s protective effects against sepsis are lost upon pharmacological inhibition or genetic disruption of LC3 in experimental settings.

## 8. Future Research Directions

A significant advantage of endogenous peptides lies in their minimal risk of immunogenicity and adverse reactions compared to synthetic drugs. Despite their promise, the clinical development of short peptides faces several major hurdles, such as poor stability in vivo due to enzymatic degradation and rapid clearance [5]. Thus, structural modifications (such as peptide cyclization or PEGylation) are essential to improve their half-life. Similarly, targeted delivery strategies, such as fusion with macrophage-targeting Fc-receptor-binding motif or encapsulation within nanoparticles and liposomes, can minimize off-target effects and increase their therapeutic concentrations at disease sites [5].

As previously mentioned, dermcidin has emerged as a key regulator of innate immunity, possessing both direct bactericidal activity [6,8] and immunomodulatory properties [1,9,17,24,32,36]. Its bactericidal action, mediated by membrane disruption, pore formation, and enzyme inhibition [8,12,33,34,35], effectively targets both Gram-positive and Gram-negative bacteria, making it a potential therapeutic agent against drug-resistant infections. Beyond direct killing, pro-DCD also modulates innate immune responses [1,9,17,24,32,36], interacting with various cell surface receptors like epidermal growth factor receptor (EGFR) [17] and suppression of tumorigenicity 2 (ST2) [36], potentially activating signaling pathways like toll-like receptor 4 (TLR4) and stimulator of interferon genes (STING) [52,53]. This modulation allows pro-DCD to fine-tune inflammation, preventing excessive responses while combating infections. Further research into these receptor interactions and signaling pathways will elucidate the full scope of pro-DCD’s immunomodulatory mechanisms and its therapeutic potential.

## 9. Conclusions

Our identification of pro-DCD as an inducible protective protein highlights the therapeutic potential of pro-DCD-C34S derivatives against lethal microbial infections. Therefore, advancing the development and clinical translation of pro-DCD-C34S derivatives for bacterial infections has become a promising possibility. With advancements in peptide engineering, targeted delivery, and preclinical validation, endogenous peptides capable of modulating innate immunity hold great promise as novel therapeutics for inflammatory diseases.

## Abbreviations

The following abbreviations are used in this manuscript:

ALT	Alanine Aminotransferase
AMP	Antimicrobial Peptide
AST	Aspartate Aminotransferase
BM-MSCs	Bone Marrow-derived Mesenchymal Stromal Cells
C	Cysteine
CLP	Cecal Ligation and Puncture
DCD	Dermcidin
EGFR	Epidermal Growth Factor Receptor
G-CSF	Granulocyte Colony Stimulating Factor
IL-6	Interleukin 6
IgG	Immunoglobulin G
KC	Keratinocytes-derived Chemokine
LC3	microtubule-associated protein 1A/1B-light chain 3
LPS	Lipopolysaccharide
MCP-1	Monocyte Chemoattractant Protein 1
MIP-2	Macrophage Inflammatory Protein-2
pAbs	Polyclonal Antibodies
PBMCs	Peripheral Blood Mononuclear Cells
PEG	PEGylation
S	Serine
ST2	Suppression of Tumorigenicity 2
sTNFRI	Soluble Tumor Necrosis Factor Receptor I
